# The Leaf Adaxial-Abaxial Boundary and Lamina Growth

**DOI:** 10.3390/plants2020174

**Published:** 2013-03-26

**Authors:** Miyuki Nakata, Kiyotaka Okada

**Affiliations:** National Institute for Basic Biology, Nishigo-naka 38, Myodaiji, Okazaki, Aichi 444-8585, Japan

**Keywords:** leaf development, the adaxial-abaxial boundary, lamina growth, leaf margin, auxin, the WOX family transcription factor

## Abstract

In multicellular organisms, boundaries have a role in preventing the intermingling of two different cell populations and in organizing the morphogenesis of organs and the entire organism. Plant leaves have two different cell populations, the adaxial (or upper) and abaxial (or lower) cell populations, and the boundary is considered to be important for lamina growth. At the boundary between the adaxial and abaxial epidermis, corresponding to the margin, margin-specific structures are developed and structurally separate the adaxial and abaxial epidermis from each other. The adaxial and abaxial cells are determined by the adaxial and abaxial regulatory genes (including transcription factors and small RNAs), respectively. Among many lamina-growth regulators identified by recent genetic analyses, it has been revealed that the phytohormone, auxin, and the WOX family transcription factors act at the adaxial-abaxial boundary downstream of the adaxial-abaxial pattern. Furthermore, mutant analyses of the *WOX* genes shed light on the role of the adaxial-abaxial boundary in preventing the mixing of the adaxial and abaxial features during lamina growth. In this review, we highlight the recent studies on the dual role of the adaxial-abaxial boundary.

## 1. Introduction

The “boundaries” between two different cell populations play a role as the center of many developmental events. In animal development (e.g., embryonic segmentation, vertebrate somite formation, brain development and *Drosophila* wing development), boundary formation is triggered by the juxtaposition of two different cell populations expressing different sets of regulatory genes at an early developmental stage, and cells located at the boundary between two different cell populations obtain special characteristics and express specific regulatory genes during boundary formation [1,2,3,4,5,6,7,8]. These special characteristics and/or specific regulators contribute to both organizing morphogenesis and preventing the intermingling of the different cell populations to maintain a sharp and straight boundary, despite perturbations due to cell proliferation and tissue deformation [3,5,7,9,10,11,12,13]. Finally, the boundary cells are differentiated into specific structures that physically separate two independent structures or two different sides of a single organ.

**Figure 1:**
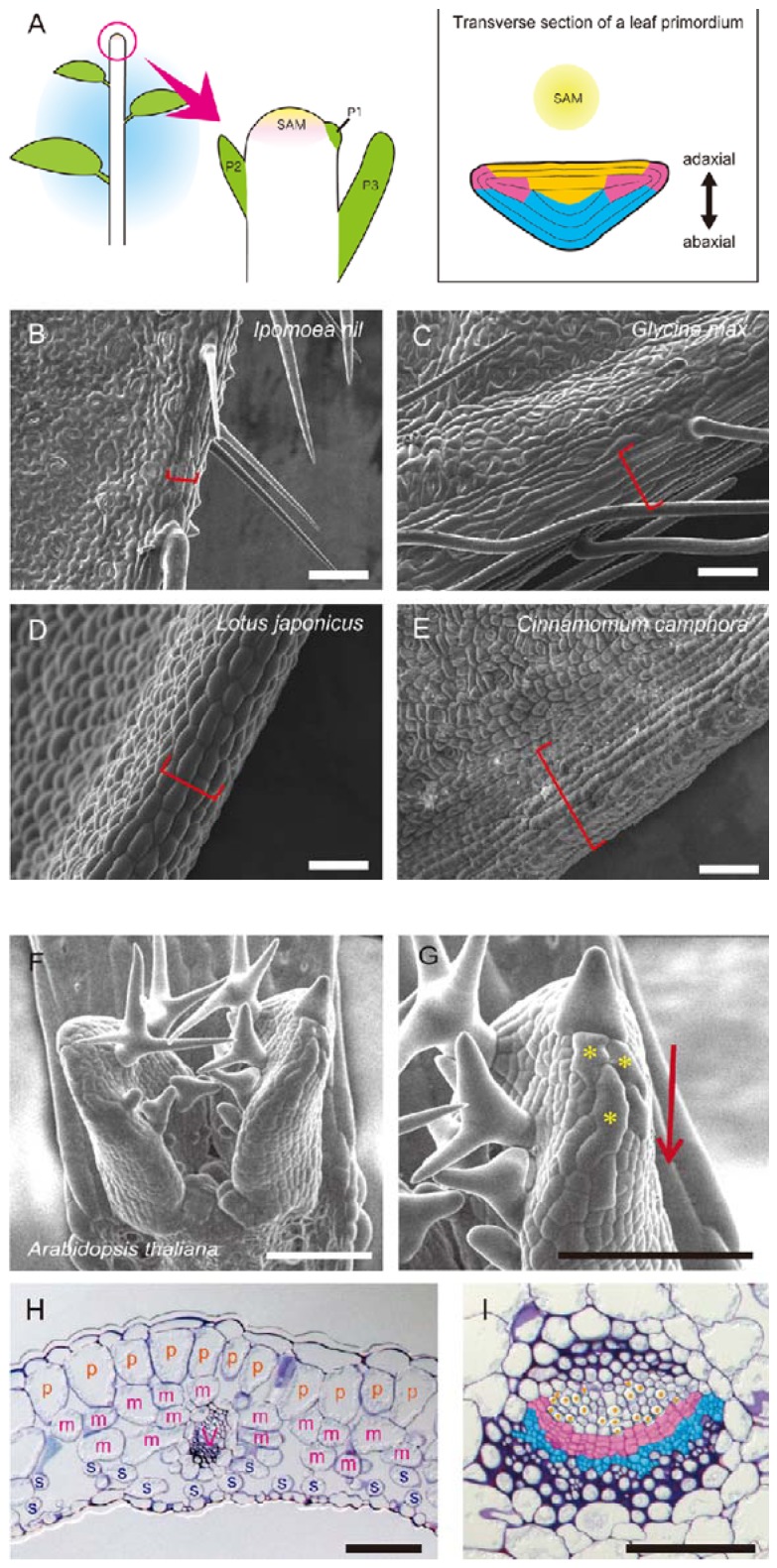
The schematic illustrations of shoots and photos of leaf tissues. (**A**) The schematic illustrations of shoots and a transverse section of leaf primordia. The shoot apical meristem is shown in yellow with “SAM” (shoot apical meristem); leaves are shown in green and the adaxial; middle and abaxial domains of leaf primordia are colored in orange, pink and cyan in the right column, respectively. P1, P2, P3 are the youngest, second and third leaf primordia, respectively. (**B**–**E**) Scanning electron micrograph of marginal cell files of leaves of *Ipomoea nil* (**B**), *Glycine max* (**C**), *Lotus japonicus* (**D**) and *Cinnamomum camphora* (**E**). Red lines show the range of the marginal cell files. (**F**–**G**) Scanning electron micrograph of *Arabidopsis thaliana* developing leaves. Asterisks show elongating margin cells; and an arrow shows the direction of the margin cell differentiation. (**H**) Transverse section of a mature leaf of *A. thaliana*. Palisade cells (p) and spongy cells (s) are observed in the adaxial (upper) and abaxial (lower) sides, respectively. In the region between palisade cells and spongy cells, middle mesophyll cells having the intermediate feature (m) are observed. Vascular cells (**V**) are also formed at the site between the adaxial and abaxial regions. (**I**) Transverse section of a vascular bundle of a mature leaf of *A. thaliana*. Xylem (shown as orange dots) and phloem (colored in cyan) are developed in the adaxial and abaxial regions inside the vascular bundle, respectively. Procambial cells (shown in pink) are formed between xylem cells and phloem cells. Scale bars, 100 µm.

In plant development, the boundaries and boundary-specific genes between two adjacent organs are well known to be important for organ separation (e.g., the boundaries between cotyledon-cotyledon, leaf-leaf, leaflet-leaflet, leaf-meristem, stem-stem, floral organ-floral organ and root-root, *etc*.) [14,15,16,17]. Additionally, the boundaries between two different sides of a single organ are considered to be important for the morphogenesis of lateral organs, which include leaves and floral organs [17,18,19,20,21]. In leaf development, the difference in gene expression level between the adaxial and abaxial sides plays an important role in cell differentiation pattern and lamina growth. The adaxial side is close to the shoot apical meristem (SAM), and the abaxial side is far from the SAM, at the early stage of primordia of lateral organs (Figure 1A). The adaxial and abaxial sides correspond to the upper and lower sides of the leaf and to the inner and outer sides of the floral organs, respectively. Lateral organs have two cell populations that have different histological features on the adaxial and abaxial sides. The adaxial and abaxial cell populations in mature organs derive from those expressing the adaxial- and abaxial-specific regulatory genes in lateral organ primordia. Because loss-of-function mutants lacking the adaxial-abaxial polarity make the organs filamentous, the adaxial-abaxial boundary is considered to be an important site for lateral organ morphogenesis, especially the lamina growth of the leaf [18,22,23].

In this review, we focus on the adaxial-abaxial boundary and lamina growth, primarily in the simple leaves of angiosperms. We introduce the following topics: (1) the structural adaxial-abaxial boundary, (2) the developmental processes of lamina growth and (3) the recent advances in understanding the regulation of lamina growth downstream of the adaxial-abaxial pattern generation. Finally, recent studies have revealed the mechanism that maintains the adaxial-abaxial pattern and the boundary during lamina growth; thus, we highlight the roles of lamina-growth regulators, especially the *WUSCHEL-RELATED HOMEOBOX* (*WOX*) family genes, in preventing the mixing of adaxial and abaxial features to maintain a proper boundary.

## 2. Histological Aspects of the Adaxial-Abaxial Boundary of the Leaf

In mature leaves of land plants, the adaxial and abaxial cells have different features and the difference is generally optimized to their biological function (e.g., optimized photosynthesis and resistance to drought stress). The adaxial and abaxial epidermal cells have different complexities and different cuticular thicknesses. The adaxial palisade mesophyll cells are oblong in shape, densely packed and contact with each other, while the abaxial spongy mesophyll cells are uneven in shape and have air spaces between them. The distribution of trichomes is imbalanced between the adaxial and abaxial surfaces, and the number of stomata on the abaxial surface is generally greater than on the adaxial surface. In vascular bundles, xylem and phloem are differentiated on the adaxial and abaxial sides, respectively.

Two middle mesophyll layers and the margin of the leaf primordia are located in the region between the adaxial and abaxial sides (which we termed “the middle domain”), which corresponds to the adaxial-abaxial boundary, based on its position (Figure 1A). The leaf margins have several types of specific structures: margin cell files, hydathodes, margin-specific hairs and stipules. Elongated margin cells are aligned along the leaf margin to form several cell files. Elongated margin cell files have been observed in many flowering plants, including *Nicotiana tabacum* [24], *Nicotiana sylvestris* [25], *Arabidopsis thaliana* [26,27], *Medicago truncatula* [28], *Ipomoea nil* (Figure 1B), *Glycine max* (Figure 1C) and *Lotus japonicus* (Figure 1E). Even in *Cinnamomum camphora*, one of the basal angiosperms (Magnoliids) [29], although non-elongated oblong cells occupy the region neighboring the margin, the cells lie on many straight lines along the margin (Figure 1E). Elongated margin cells are localized at the adaxial-abaxial boundary of the epidermis. The differentiation precedes adaxial and abaxial epidermal cells (except for trichomes) and proceeds in a basipetal manner (Figure 1F,G) [27,30,31,32]. In addition, hydathodes are scattered at the leaf margin to create gaps of elongated margin cells [27], and stipules are formed at the lateral base of the leaf primordium in Brassicaceae. Taken together, the marginal structures create a structural boundary to separate the adaxial and abaxial cell populations from each other during explosive blade outgrowth.

In the mesophyll layers, the adaxial and abaxial cell populations are not completely separated from each other in terms of the histological aspect. In the internal layers of the middle domain, vascular bundles and mesophyll cells with an intermediate character between palisade and spongy cells are formed (Figure 1H), and the cells located in the domain do not appear to have a role as a structural boundary. In vascular bundles, procambium cells are maintained between the adaxial and abaxial regions, including xylem and phloem, respectively, and block contact between the two regions (Figure 1I). In conclusion, epidermis and vascular bundles create the structural boundaries between the adaxial and abaxial cell populations, but mesophyll does not.

## 3. The Regulators of the Adaxial-Abaxial Patterning of the Leaf

**Figure 2:**
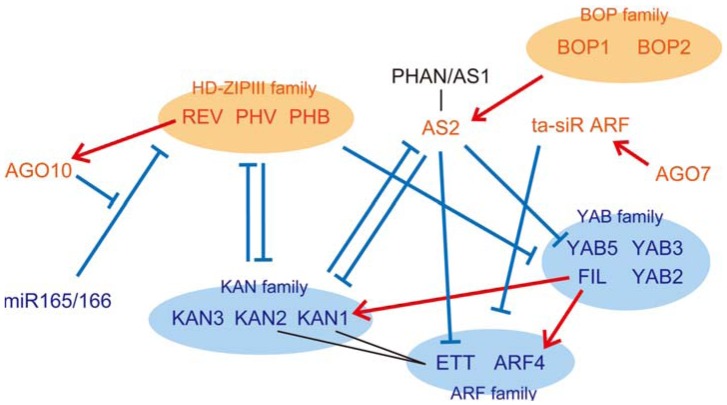
Reported interactions among the adaxial and abaxial regulators. The adaxial-specific regulators are shown in orange characters. The abaxial-specific regulators are shown in blue characters. PHAN/AS1 shown in black character is expressed broadly in leaf primordia. Red arrows show positive regulations, cyan wedges show negative regulations and black bars show direct interaction between two regulatory proteins.

The difference between the adaxial and abaxial sides begins to appear at the early stage of leaf development as a distinct pattern of gene expressions. The proper adaxial-abaxial pattern is established, refined and maintained in accordance with a complicated regulatory network involving many regulators (Figure 2). The adaxial- or abaxial-specific genes, belonging to five different families of transcription factors, have been identified as the major regulators specifying the adaxial or abaxial regions: in the case of *A. thaliana*, three homeodomain-leucine zipper (*HD-ZIPIII*) family genes (*PHABULOSA* (*PHB*), *PHAVOLUTA* (*PHV*) and *REVOLUTA* (*REV*)) [33,34,35,36] and the *ASYMMETRIC LEAVES2* (*AS2*) gene [37,38,39,40] have been identified as adaxial regulators and four *YABBY* (*YAB*) family genes (*FILAMENTOUS FLOWER* (*FIL*), *YAB3*, *YAB5* and *YAB2*) [26,41,42], three *KANADI* (*KAN*) family genes (*KAN1*, *KAN2* and *KAN3*) [43,44,45] and two *AUXIN RESPONSE FACTOR* genes (*ETTIN* (*ETT*)/*ARF3* and *ARF4*) [46,47] as abaxial regulators. PHANTASTICA (PHAN)/ASYMMETRIC LEAVES1 (AS1), which is a MYB domain transcription factor, binds to AS2 and coordinates adaxial specification together with AS2, although gene expression is detected in the broad region of young leaf primordia [37,40,48]. These regulators, except for *PHAN*/*AS1*, are specifically expressed in the adaxial or abaxial regions, and their mutual regulation contributes to the establishment and maintenance of a clear adaxial-abaxial pattern. The *HD-ZIPIII* genes directly or indirectly downregulate the *FIL* genes [42] and antagonize the *KAN* family genes [43,44,49,50]. *KAN1* directly represses *AS2* expression, and *AS2* indirectly represses *KAN1* expression [51]. *AS2* negatively regulates *ETT, KAN2* and *YAB5* [37]. *FIL* directly upregulates *KAN1* and *ARF4* [52]. Furthermore, KAN1 binds to ETT in a yeast system [53], implying that KAN1 coordinates the abaxial identity together with ETT. In addition, the BTB/POZ domain-containing transcriptional coactivator, BLADE ON PETIOLE (BOP) proteins, which are expressed in the proximal, adaxial region of leaf primordia, are involved in the adaxial-abaxial patterning and directly activate *AS2* expression [54]. Overexpression of *BOP1* slightly increases *PHB* expression and slightly decreases *FIL* and *KAN1* expression in the petiole region [55]. These regulations demonstrate that the regulators of the same side work in a coordinated manner, while those of the opposite side are antagonists.

Two types of small RNAs, the 21-nt micro RNA (miRNA), miR165/166, and the 24-nt trans-acting small interfering RNA (ta-siRNA), ta-siR ARF, are also critical for pattern formation of the adaxial and abaxial regulators [19,56]. DICER-LIKE1 (DCL1), HYPONASTIC LEAVES1 (HYL1) and SERRATE (SE) are involved in the generation and maturation of the miRNAs [57,58]. In ta-siR ARF biogenesis, transcripts from the *TAS3* loci are cleaved by the function of miR390 and AGO7/ZIPPY; the cleaved *TAS3* transcripts are converted to double stranded RNA (dsRNA) by SUPPRESSOR OF GENE SILENCING3 (SGS3) and RNA-DEPENDENT RNA POLYMERASE6 (RDR6) and the dsRNA is cleaved to a 24-nt dsRNA in a DICER-LIKE4 (DCL4)-dependent manner [57,58]. Through the function of HUA ENHANCER1 (HEN1) and ARGONAUTE1 (AGO1), miR165/166 cleaves transcripts of the target genes, *HD-ZIPIII*s, in the abaxial region [59,60], whereas ta-siR ARF degrades transcripts of *ETT* and *ARF4* in the adaxial region [46,61,62,63,64,65] (Figure 2). Two of the AGO proteins contribute to the restricted distribution of the small RNAs. The AGO10/PINHEAD/ZWILLE protein is specifically expressed in the SAM and the adaxial region of lateral organs [66], binds to miR165/166 and prevents their functioning, thereby contributing to the adaxial-specific accumulation of *HD-ZIPIII* transcripts [67] (Figure 2). *AGO10* expression is upregulated by *REV*, one of the *HD-ZIPIII* genes [68] (Figure 2). The expression of *AGO7* is restricted to the adaxial region to limit the distribution of ta-siR ARF [46] (Figure 2).

In addition to small RNAs, small proteins containing a leucine zipper domain similar to HD-ZIPIII (ZPR) modulate the negative feedback regulation of HD-ZIPIII: the HD-ZIPIII protein REV activates *ZPR* transcription, and the ZPR proteins bind to the leucine zipper domain of HD-ZIPIII to decrease the activity of HD-ZIPIII [69]. Furthermore, the roles of several housekeeping genes, including ribosomal proteins (RPL4D, PGY3/ASYMMETRIC LEAVES 1/2 ENHANCER6 (AE6)/RPL5A, RPL5B, PGY2/RPL9C, PIGGYBACK1 (PGY1)/RPL10aB, RPL24B, RPL27aC, AE5/RPL28A (AE5), RPS6A and RPS21B), histone deacetylases, the proteasome pathway, plastid-related genes and elongator complexes, have also been reported in adaxial-abaxial patterning [70,71,72,73,74,75,76,77,78]. The overaccumulation and exogenous application of succinic semialdehyde (SSA), a metabolite in the GABA shunt, disturbs the adaxial-abaxial polarity, suggesting that the balance between the adaxial and abaxial regulators at the early stage of leaf primordia is affected by the amount of the metabolite SSA [79]. The regulatory network for the establishment and maintenance of the adaxial-abaxial pattern is not yet well understood, because the network consists of a number of factors, including transcription factors, small RNAs, the AGO proteins, small proteins, housekeeping genes and SSA, and the interactions among the factors are complicated. Work using a systems biology approach in the sepal primordium has been reported [80], and the entire picture of the network will be elucidated in the near future.

## 4. Lamina Growth and Its Regulators

**Figure 3:**
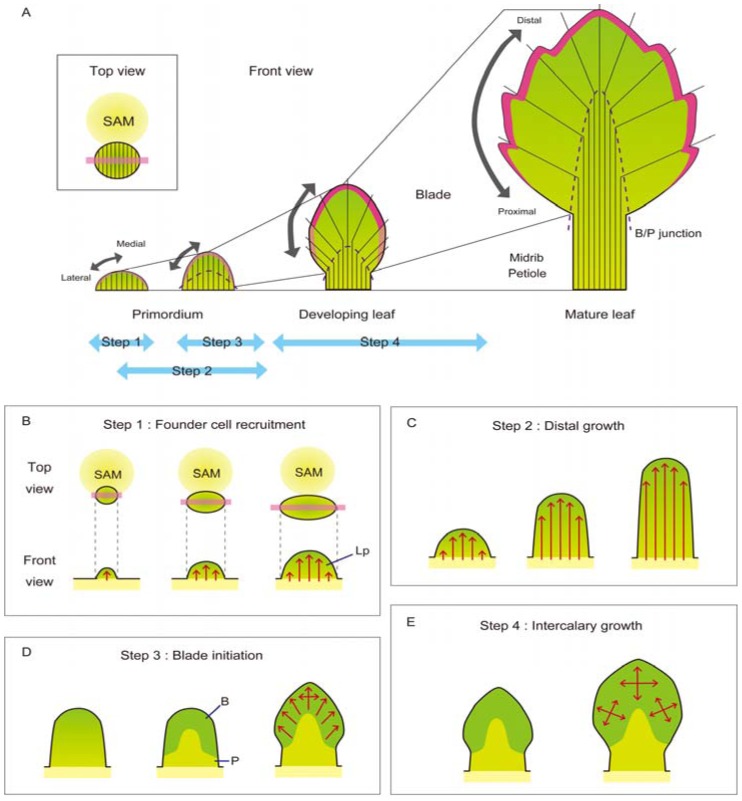
Schematic views of leaf development. (**A**) The overview of dicot leaf development. Leaf primordia are illustrated in the top view and the front view, and black lines show the borderlines between the individual sectors sharing a common origin in the incipient primordium. Light pink shows the region of indeterminate margin cells, and magenta shows the region of differentiated margin cells. Dashed lines show the B/P junction. (**B**–**E**) Schematic views of four steps during leaf development. First, the initiation of leaf primordia (indicated as “Lp”) starts through the recruitment of the founder cells from the peripheral zone of the SAM, (**B**) and then, the initiated leaf primordium grows predominantly in the distal direction (**C**). Subsequently, the blade region (indicated as “B”) the petiole region (indicated as “P”) are specified on the basis of the distance from the margin, (**D**) and finally, the leaf blade expands towards both the distal and lateral directions (**E**). Red arrows in (**B**–**E**) show the direction of leaf growth.

Leaf morphogenesis is accomplished through four processes: founder-cell recruitment from the peripheral zone of the SAM (Step 1), distal growth (Step 2), blade initiation at the site neighboring the margin (Step 3) and intercalary growth throughout the blade region (Step 4) [24,81,82,83,84]. Figure 3 shows the schematic perspective view of leaf morphogenesis (Figure 3A) and illustrations of each step (Figure 3B–E).

The initiation of lateral organs, including the leaves, arises at a site in the peripheral zone (PZ) of the SAM (Figure 3A,B) and is triggered by the accumulation of phytohormone auxin [85] and the alteration of cell-wall behavior [86,87,88,89]. In the area neighboring the auxin maxima in the PZ, a group of founder cells obtain a fate as organ primordia. The number of founder cells was calculated to be 20–30 cells in a leaf of A. *thaliana* [90,91], 100–150 cells in the tobacco leaf (Poethig and Sussex, 1985a; Steeves and Sussex, 1989) and approx. 250 cells in *Zea mays* leaves [81,82] based on clonal analyses. The eudicot leaf primordium, including *A. thaliana* and *N. tabacum*, is initiated as a peg-like outgrowth, whereas the monocot leaf primordium, including *Z. mays*, is initiated as a ring-shaped outgrowth surrounding the SAM [81]. Primordial initiation of a simple leaf accompanies a cessation of the expression of the meristem-specific genes, *i.e.*, the *KNOTTED-LIKE HOMEOBOX* (*KNOX*) family genes [92,93,94,95,96,97,98]. Repression of *KNOX* expression in leaf primordia is triggered and/or maintained by leaf primordium-specific factors (e.g., auxin, *AS1*, *AS2*, *FIL/YAB* and *BOP*) [55,94,99,100,101,102,103]. The distribution of auxin is also known to be a crucial factor for determining the range of founder-cell recruitment in both eudicot and monocot species [85,104]. In the case of *Z. mays* leaves, the functioning of the *NARROW SHEATH* (*NS*) genes, which encode plant-specific homeobox transcription factors that belong to the WUSCHEL-RELATED HOMEOBOX (WOX) family, is also required for normal recruitment of leaf founder cells from the lateral region and for repression of *KNOX* expression [105,106,107]. In *A. thaliana*, overexpression of *WOX1*, one of the *WOX* family genes related to *NS* (but not its orthologous gene), is also reported to induce a defect in the SAM [108].

Subsequently, the initiated primordium grows in the distal direction (Figure 3C). In the case of the sepal primordium in *A. thaliana*, the anisotropic distal growth is an important factor for the transition of the fate from the meristem to the primordium, as well as accelerated cell division, cell enlargement and an increase in cell-size heterogeneity [109]. The fate transition, regulated by the JAGGED (JAG) transcription factor, is not essential for organ initiation, but is important for the primary growth of organs [109]. During leaf development, the *JAG* gene is expressed [110,111]. and the *NUBBIN* gene, a gene homologous to *JAG*, acts redundantly with *JAG* in leaf development [112]. Hence, the fate transition regulated by JAG may also be important for the beginning of distal growth during leaf development. In eudicot leaves, lamina growth begins a little later than distal growth [81] (Figure 3A,D). In early primordia, a high frequency of cell division is detected at the region neighboring the margin [83,113,114]. and the periclinal division of the third layer from the surface is specifically found in the region neighboring the margin [115], corresponding to the onset of the blade outgrowth. Clonal analyses, showing the fan-shaped sectors in eudicot leaves [81,82,90,91], and the difference between the distal and basal cells in cell shape and alignment in early leaf primordia [116] support a model in which the blade region in mature leaves corresponds to the part neighboring the margin in early leaf primordia (the green area in Figure 3D). The junction between the blade region and the petiole region (the B/P junction) is the site expressing the *CYCLIN D4;2* gene, presumably involved in the cell cycle, and can act as a source of both blade and petiole cells [116]. The formation of the B/P junction requires the function of the *BOP* genes [116], which are specifically expressed in the petiole region and reduce the expression level of the meristem-specific *KNOX* genes [101] and of the lamina-specific *JAG* gene [117]. The formation of the B/P junction might be a key step for determining the blade area at the blade-initiation stage. For the blade initiation of eudicot leaves, the *WOX* family genes, including the *A. thaliana PRESSED FLOWER* (*PRS*) gene, which are orthologous to the *Z. mays NS* genes and the *WOX1* genes (*N. sylvestris LAMINA1* (*LAM1*), *A. thaliana WOX1*, *Petunia hybrida MAEWEST* (*MAW*), *M. truncatula STENOFOLIA* (*STF*) and *Pisum sativum LATHYROIDES* genes), are reported to play a key role [25,28,113,115,118,119]. The *lam1* mutant of *N. sylvestris* has leaves that lack the blade region, and the *prs wox1* double mutant of *A. thaliana*, the *maw* mutant of *P. hybrida* and the *stf* mutant of *M. truncatula* have narrow leaves. In *prs wox1*, the frequency of cell division at the marginal region of early leaf primordia decreases to the same level as at the central region [113]. The accumulation of *PRS* and *LAM1*/*WOX1* transcripts can be detected at the margin of early primordia [28,113,119,120]. Thus, it is suggested that *PRS* and *LAM1*/*WOX1* increase the rate of cell division and stimulate the periclinal division in the region neighboring the margin to contribute to blade initiation.

After blade initiation, the expansion by both cell division and cell differentiation occurs in the entire blade region, called “intercalary growth” (Figure 3E). The transition from the cell-division stage to the cell-differentiation stage proceeds in a basipetal manner [82,83,121], and the forefront of the transition is named the “arrest front” [121]. The fan-shaped sectors in clonal analyses [81,82,90,91] indicate that the medial and lateral parts of the leaf primordia correspond to the distal and proximal parts of the leaf blade in mature leaves, respectively (Figure 3A). Namely, the basipetal transition from the cell-division stage to the cell-differentiation stage in the leaf blade of developing leaves can be interpreted as the medial-to-lateral transition in early leaf primordia. The analysis of growth dynamics by time-lapse imaging supports the deformation of the growth axis during blade expansion [122]. Recent studies have identified many regulators affecting the rate or duration of cell division during intercalary growth. The rate of cell division is positively regulated by *FUGU2*, *FUGU5* and *ERECTA* [123] and negatively regulated by *SPATULA* and *ROTUNDIFOLIA4* [124,125,126]. In contrast, the duration of cell proliferation is prolonged by *AINTEGUMENTA* (*ANT*) [127], *AUXIN-REGULATED GENE INVOLVED IN ORGAN SIZE* (*ARGOS*) [128] and its related genes (*ARGOS-LIKE* (*ARL*) or *GAN SIZE RELATED1* (*OSR1*)) [129], the *GROWTH-REGULATING FACTOR* (*GRF*) family genes [130], the *ANGUSTIFOLIA3* (*AN3*)/*GRF-INTERACTING FACTOR* (*GIF*) family genes [130,131,132], the microRNA miR319 [133,134], the *KLUH* (*KLU*)/*CYP78A5* gene [135] and the *STRUWWELPETER*/*MED14* gene [136], whereas the duration of cell proliferation is shortened by the microRNA miR396, which targets some of the *GRF* family genes [137], the *PEAPOD1* (*PPD1*) and the *PPD2* genes [138], the *TCP* family genes targeted by microRNA miR319 [121,133], the *DA1* gene and its related genes (*DAR*) [139] and the *MED25*/*PFT1* gene, which encodes the mediator complex subunit 25 [140]. Of these genes, *AN3* and *KLU* can affect cell behavior in a non-cell autonomous manner [135,141,142], and the non-cell autonomous effect may be important for orchestration among the blade cells adjacent to each other during intercalary growth. The *WOX1* orthologous genes also act in this step, because these genes are expressed in the middle mesophyll layers between the adaxial and abaxial sides throughout the leaf blade [28,113,119] and upregulate the *KLU* expression at the leaf margin [113]. In addition, phytohormones are also important for lamina expansion. Auxin is one of the most important regulators of blade expansion out of phytohormones. Auxin accumulation is required for the outgrowth of the tip of the margin serrations [100,143]. The quadruple mutant of *YUCCA* (*YUC*) family genes, which are auxin biosynthetic genes [144,145,146], *i.e.*, the *yuc1 yuc2 yuc4 yuc6* quadruple mutant, forms very narrow leaves [147]. Furthermore, treatment with exogenous auxin stimulates the expression of *ARGOS*, which induces *ANT* expression and may contribute to lamina growth [148], consistent with the model in which blade expansion is positively regulated by auxin. It has also been reported that treatment with exogenous auxin prevents blade expansion [149], most likely through inhibition of cell expansion [150], implying that the proper amount and distribution of auxin are important for promoting blade expansion. In addition to auxin, brassinosteroid, another phytohormone, is reported to be important for both cell proliferation and cell expansion during lamina growth [151,152,153,154,155].

The recruitment of founder cells (Step 1) appears to be crucial for the lamina growth of monocots, although blade initiation (Step 3) and intercalary growth (Step 4) are the most important steps for lamina growth in eudicot species. In the clonal analysis of *Z. mays* leaves, clonal sectors are more thread-like than fan-like in shape [81]. Additionally, mutations of the *NS* genes resulted in the failure to recruit founder cells of the lateral region to form narrower blades and sheaths of mature leaves [105,106]. It is also reported that the double mutant of *Oryza sativa NARROW LEAF2* (*NAL2*) and *NAL3* orthologous to *Z. mays NS* genes shows the defects of the leaf lateral region, similar to *Z. mays ns* mutant [156].

## 5. The Regulation of Lamina Growth and Margin Formation at the Adaxial-Abaxial Boundary

As we described above, adaxial-abaxial polarity is required for lateral organ morphogenesis, especially lamina growth and margin formation of the leaf. In surgical experiments, an incipient leaf primordium that was physically separated from the shoot apical meristem (SAM) became a filament-like structure lacking adaxial-abaxial polarity, indicating that both the establishment of adaxial-abaxial differentiation and the onset of the blade initiation require connection with the SAM in *Solanum tuberosum L.* and *S. lycopersicum* [81,157]. Additionally, Waites and Hudson reported an *Antirrhinum majus phan* mutant in which the adaxial cell fate was weakened or missing [23]. In the *phan* mutant, two opposite phenotypes were observed in terms of lamina growth: (1) no or decreased growth of the leaf blade and (2) adventitious outgrowths at the ectopic boundary of the adaxial- and abaxial-like compartments on the adaxial surface [23]. Similar defects were also found in several mutants in *Z. mays* [22,158,159], *A. thaliana* [33,39,42,43,44,45,47,79,160], *N. sylvestris* [161] and *I. nil* [162], as well as in *A. majus* [114]. In eudicots, even plants lacking adaxial-abaxial polarity display leaf initiation from the SAM (Step 1) and distal growth of leaf primordia (Step 2). Therefore, it is hypothesized that the juxtaposition of the adaxial and abaxial cell populations is required for proper lamina growth, consisting of lamina initiation (Step 3) and intercalary growth (Step 4), at the adaxial-abaxial boundary [18,21,163]. In addition to lamina growth, it has been suggested that development of margin-specific structures is affected by the malfunction of the adaxial-abaxial polarity. In adaxial- or abaxial-deficient mutants, a filamentous or narrow leaf phenotype is accompanied by the deletion of margin-specific structures, whereas adaxial or abaxial protrusions have margin-like structures, *i.e.*, elongated cells, hydathode-like structures and stipules [32,41,42,43,44,160]. As described above, margin-specific structures are formed at the adaxial-abaxial boundary of the epidermis. In conclusion, it has been suggested that both lamina growth and margin formation are stimulated by the juxtaposition of the adaxial and abaxial cell populations.

Of the adaxial and abaxial factors, the *YAB* family genes, especially *A. thaliana FIL/YAB1* and *YAB3* and *A. majus GRAMINIFOLIA* (*GRAM*), act in lamina growth and margin formation in addition to adaxial-abaxial patterning [41,42,44,114]. The *YAB* family genes show specific expression in the abaxial region of lateral organ primordia during lamina growth in eudicots [26,41,42,44,164], whereas the expression of the *YAB* genes is detected in the broad region at the leaf-initiation stage [164,165]. Furthermore, the expression of *YAB* family genes is restricted into the middle mesophyll layers and the leaf margin in monocot *Z. mays* and *O. sativa* leaves, rather than the abaxial region [166,167]. The expression of the *YAB* genes is correlated with lamina growth in both *A. thaliana* and *S. tuberosum* [44]. The *YAB* family genes are ectopically expressed at the abaxial protrusions of the *kan1 kan2* leaves [44]. The mutation of *FIL* and/or *YAB3* suppresses formation of the abaxial protrusions of *kan1 kan2* leaves [44] and of ectopic lamina growth of *bop1 bop2* leaf petioles [102]. These findings suggest that the *YAB* genes play a role in lamina growth. However, overexpression of the *YAB* genes prevents blade outgrowth, perturbs margin formation and results in the arrest of the SAM [26,42]. In addition, the *YAB* genes need to downregulate the expression of the meristem-specific genes, including *KNOX* and *WUSCHEL* (*WUS*) [42,168], and upregulate the expression of the *TCP* family genes to act as a negative regulator of intercalary growth of the leaf lamina [41]. Furthermore, the *YAB* genes are required not only for abaxial features, but also for adaxial features and adaxial *PHB* expression [169,170]. Thus, it can be concluded that the *YAB* family genes not only promote lamina growth, but also cause the foliar characteristics of the leaf primordium.

Of the lamina-growth regulators, recent studies based on *A. thaliana* reveal the two candidates that mediate lamina growth and margin formation at the adaxial-abaxial boundary downstream of adaxial-abaxial patterning. The first candidate is the phytohormone, auxin. Based on the expression of the auxin-response reporter DR5 markers, auxin is accumulated at the leaf tip, hydathodes and stipules and in the preprocambial cells in leaf primordia [171,172,173]. The DR5 marker pattern and the polar localization of the auxin efflux carrier PIN1 protein indicate that auxin (1) flows along the leaf margin to form several auxin maxima, (2) floods into the inner mesophyll layers from the auxin maxima and (3) reaches the midrib [171,173,174]. In this manner, the trajectory of auxin in leaf primordia is restricted to the middle domain. The number and the position of auxin maxima affects lamina growth, as well as the number and the position of the hydathodes or the margin gaps, and formation of auxin maxima is regulated by the function of the adaxial-specific *AS2* gene and its coregulator, *PHAN/AS1* [27]. In addition, the auxin biosynthetic *YUC* family genes (*YUC1*, *YUC2* and *YUC4*) are expressed in the early leaf primordia, including the region neighboring a hydathode at the margin and/or the middle mesophyll, and expression is dependent on the adaxial-abaxial pattern [32]. The expression of these *YUC* genes is ectopically detected at the adventitious protrusions on the adaxial or abaxial surface of leaves in the abaxial-deficient *kan1 kan2* mutant and the adaxial-deficient *as2 rev* mutants [32]. Mutations of the *YUC* genes and inhibition of polar auxin transport by NPA treatment suppress the adventitious protrusions in both *kan1 kan2* and *as2 rev* mutant backgrounds [32]. According to these findings, it is suggested that the amount and distribution of auxin mediate blade outgrowth at the adaxial-abaxial boundary downstream of the adaxial-abaxial pattern. The adaxial-specific *REV* gene and the abaxial-specific *KAN1* gene positively and negatively regulate auxin biosynthetic genes (*i.e.*, *TAA1* and *YUC5*), respectively [175]. These data are evidence of the direct regulation of the auxin level by the adaxial and abaxial regulators.

The second candidate genes are the *WOX* family genes, including *PRS* and *WOX1*. *PRS* and *WOX1* in *A. thaliana* and the *WOX1* orthologous gene, *STF*, in *M. truncatula* are required for both lamina growth and marginal cell fate [28,113]. *PRS* and *WOX1* in *A. thaliana* are specifically expressed in the middle domain [113,119,120,176], and similar expression patterns of the *WOX1* orthologous genes in *P. hybrida*, *M. truncatula* and *P. sativum* are observed in developing leaves [28,118,119]. The expression domain of *PRS* and *WOX1* does not overlap with and is located between the expression domains of the adaxial- and abaxial-specific regulators, except for *FIL* [113]. The expression of *PRS* and *WOX1* is detected in the broad region of the abaxial side in the *kan1 kan2* double mutant, which expresses abaxial protrusions [113]. The expressions of *PRS* and *WOX1* are indirectly and directly repressed by KAN1, respectively [113]. *WOX1* expression was decreased in the *fil yab3* double mutant, which has narrow leaves [113], and in the *prs wox1 as2* triple mutant, in which leaves are completely abaxialized [176]. Furthermore, ectopic expression of *WOX1* in the abaxial region induces adventitious outgrowth with margin-like structures on the abaxial surface of the leaf similar to *kan1 kan2* [113]. These findings strongly suggest that *PRS* and *WOX1* play key roles in lamina growth and margin formation at the adaxial-abaxial boundary downstream of the adaxial and abaxial regulators.

**Figure 4:**
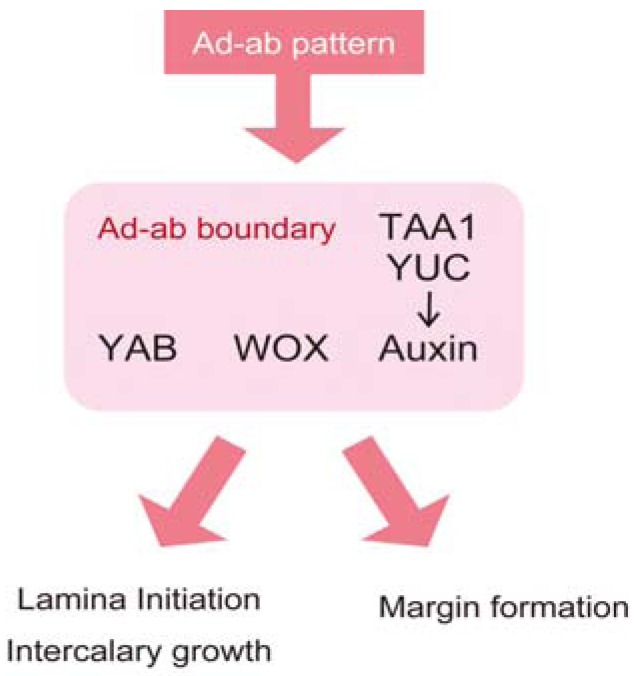
The model of the regulation of lamina growth and margin formation. The lamina-growth regulators, including YAB, WOX1 and auxin, act in lamina growth and margin formation at the adaxial-abaxial boundary downstream of the adaxial-abaxial pattern (Ad-ab pattern).

With respect to the relationship between auxin and *WOX1*, Tadege *et al*. proposed that *WOX1/LAM1/STF* affects the amount of auxin [28]. In a transcriptome analysis of an *M. truncatula stf* mutant, the alteration of the expression level of the auxin-related genes is overrepresented. In an *M. truncatula stf* mutant and an *N. sylvestris lam1* mutant, the amount of auxin is reduced compared to wild-type, whereas the overexpression of *STF* causes the overaccumulation of auxin. Although how *WOX1/LAM1/STF* affects the amount of auxin is unknown, mutation alters the accumulation pattern of metabolites in the IAA biosynthetic pathway and the metabolic pathway upstream of Trp, including sugar metabolism. The bladeless phenotype of the *N. sylvestris lam1* mutant is partly rescued by application of both auxin and cytokinin, suggesting that auxin and cytokinin are downstream factors of the *WOX1/LAM1* gene for lamina growth. Thus, *WOX1/LAM1* might facilitate the accumulation and/or the function of both auxin and cytokinin to promote lamina growth. In fact, the homologous genes that are closely related to *WOX1/LAM1* are reported to be involved in the accumulation and signaling of cytokinin; the *A. thaliana WUS* gene positively regulates cytokinin signaling through downregulation of the type-A *ARABIDOPSIS RESPONSE REGULATOR* genes [177], and the *O. sativa WOX4* gene increases the amount of cytokinin [178]. In addition to the relationship between auxin and the *WOX* genes, it has been shown that *FIL* expression overlaps the *WOX* genes in the middle domain and that *FIL* and/or *YAB3* are required for the upregulation of the *WOX1* expression [113]. Based on these findings, the model shown in Figure 4 can be proposed: (1) the expression of the auxin biosynthetic genes and of the *WOX* genes is induced at the adaxial-abaxial boundary in accordance with the adaxial-abaxial pattern, (2) the *FIL/YAB* genes upregulate *WOX1* expression and *WOX* reinforces accumulation of auxin via several metabolic pathways and (3) *FIL/YAB*, *WOX* and auxin (and most likely also cytokinin) coordinate lamina growth (*i.e.*, blade initiation and intercalary growth) and margin formation.

## 6. Maintenance of the Adaxial-Abaxial Pattern during Lamina Growth

Studies of the *WOX* family genes (*PRS* and *WOX1/LAM1/MAW*) in *N. sylvestris*, *P. hybrida* and *A. thaliana* shed light on a new role of the adaxial-abaxial boundary during adaxial-abaxial patterning [25,113,119,176]. In *N. sylvestris lam1* leaves, the marginal elongated cell files are missing from the marginal region and transform into cells having neither adaxial nor abaxial feature [25]. The margin loss and a polarity defect in the marginal region are also observed in *P. hybrida* and *A. thaliana*: the region neighboring the margin exhibits an abaxialized phenotype in a *P. hybrida maw* mutant [119], and the region consists of both adaxial-like cells and abaxial-like cells (*i.e.*, abaxial-type epidermal cells, trichomes, palisade-like cells and spongy-like cells) in the *A. thaliana prs wox1* double mutant [113]. Based on periclinal chimera analysis in *N. sylvestris*, the chimeric plant having the *lam1* defect in both the L1 and L2 layers (the L1-L2 *lam1* chimera) exhibits both margin loss and an adaxial-abaxial polarity defect in the region neighboring the margin, despite normal outgrowth of the blade [25]. These findings suggest that the *LAM1/WOX1* function in the L1 and L2 layers is important for genes that play a role in adaxial-abaxial patterning, whereas the *LAM1/WOX1* function in the L3 layer is sufficient for blade outgrowth. In the L1-only *lam1* chimera, the margin cells and adaxial-abaxial polarity are almost normal [25], implying that the L2 layer is sufficient for *LAM1/WOX1* function in the adaxial-abaxial patterning and margin formation. Based on genetic analyses, mutations of *PRS* and *WOX1* can enhance both adaxialization and abaxialization in the abaxial-deficient mutants (*kan1 kan2* and *fil yab3*) and adaxial-deficient mutants (*as2* and *rev*), respectively [113,176]. Taken together, it can be concluded that the *WOX* genes act in suppression of both adaxial- and abaxial-specific features from the L2 layer of the adaxial-abaxial boundary. Based on RNA *in situ* hybridization analysis, the expression patterns of *AS2* and *FIL* in the *prs wox1* primordia are similar to those in the wild-type primordia before blade initiation, whereas at the later stage, both expression domains expand toward the opposite side and overlap each other in the marginal region [113]. The *WOX* genes function in the downregulation of not only *AS2* and *FIL*, but also *ARF4* and *miR165A* [119,176]. The PRS and WOX1/STF proteins act as transcriptional repressors [179,180], and the WOX protein and other homologous proteins exhibit repressive activity that is interchangeable [180,181]. In conclusion, *WOX* function in the L2 layer of the adaxial-abaxial boundary represses several adaxial- and abaxial-specific regulators to maintain separation of the adaxial and abaxial features.

**Figure 5:**
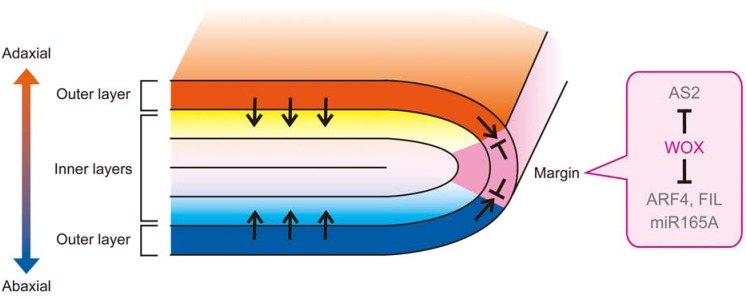
The model of the maintenance of the adaxial-abaxial pattern during lamina growth. A schematic view of the transverse section of leaf primordia during lamina growth. The adaxial and abaxial regulators, including mobile signaling factors, are expressed in the adaxial and abaxial cells of the outer layer (colored in orange and cyan), respectively and contribute to the maintenance of the identity of the inner layers in a non-cell-autonomous manner (black arrows). The effect of the mobile signaling factors reaches the margin cells, but the middle domain-specific *WOX* genes inhibit the expression of the adaxial and abaxial regulators, resulting in preventing the mixing of the adaxial and abaxial features and maintaining the adaxial-abaxial pattern (shown in a right balloon).

In the previous study, it was found that the adaxial- and abaxial-specific regulators, except for *FIL*, are expressed in one or two outer layer(s) in *A. thaliana* [37,46,113,182]. In particular, AS2, ta-siR ARF and miR165/166 are generated in the outermost layer of the adaxial or abaxial sides [37,46,182]. The regions where the small RNAs degrade their target genes are broader in leaf primordia [46,79], and the small RNAs also function in roots and/or embryogenesis in a non-cell-autonomous manner [183,184,185,186], suggesting that these small RNAs act as mobile signals. In addition, in *prs wox1* leaf primordia, the expansion of the transcriptional region of miR165A in the epidermal layer coincides with restriction of the YFP-positive region of the *miYFP-W* marker targeted by miR165/166 in both the outer and inner layers neighboring the margin [113,176]. Thus, it is possible that the adaxial-abaxial pattern of the epidermal layer may guide that of the inner layers. Taken together, we propose a hypothesis for the mechanism maintaining the adaxial-abaxial pattern and the adaxial-abaxial boundary during lamina growth. First, an initiated leaf primordium obtains adaxial-abaxial polarity based on the presumptive positional signal(s) from the SAM. Second, based on the adaxial-abaxial polarity, the adaxial and abaxial regions and the adaxial-abaxial boundary are determined. Then, the *WOX* family genes, *PRS* and the *WOX1* orthologous genes are expressed at the adaxial-abaxial boundary (or the middle domain). Fourth, the *WOX* genes repress the expression of several adaxial- and abaxial-specific regulators in the outer layers of the adaxial-abaxial boundary, corresponding to the leaf margin and prevent expansion of adaxial and abaxial domains (Figure 5). The *WOX* genes induce blade initiation and promote lamina expansion in the internal layers of the adaxial-abaxial boundary. Fifth, the adaxial-abaxial pattern of the inner layers is maintained according to the distribution of small RNAs from the outer layer during lamina growth (Figure 5). Finally, the *WOX* genes induce the differentiation of the margin-specific structures to form a structural boundary. Because the defects of the adaxial-abaxial pattern and of margin formation are also observed in the epidermal layer of *N. sylvestris lam1*, *A. thaliana prs wox1* and *M. truncatula stf* [25,28,113], the sufficiency of *LAM1/WOX1* function in the L2 layer [25] suggests that *WOX1* orthologous genes can affect neighbor cells during these two processes. Cell-to-cell movement of the WUS protein, one of the homologues closely related to WOX1, has been reported [187]. Therefore, the WOX1 protein might move between the L1 and L2 layers. This hypothesis is consistent with the fact that adaxial epidermal cells are completely separated from abaxial cells by margin-specific cells, and the separation of the adaxial and abaxial mesophyll cells is obscure.

Of the lamina-growth regulators, ANT and AN3 are also responsible for the adaxial-abaxial patterning. Mutations in *ANT* and *AN3* enhance the defects of the adaxial-abaxial pattern in *fil yab3* and *as2*, respectively [169,188]. Genetic analysis suggested that the *ANT* and *YAB* genes coordinately act as positive regulators of both genes, specifying adaxial or abaxial fates [169]. Recently, it has been proposed that *ANT* modulates auxin homeostasis through the regulation of the auxin biosynthetic *TAA1* expression with *REV* in gynoecium development [189]. Thus, *ANT* might play a role in the reinforcement of the adaxial-abaxial pattern and auxin induction at the adaxial-abaxial boundary in coordination with the adaxial and abaxial regulators. The *an3 as2* double mutant (and also the *an3 as1* double mutant) exhibit a more severe defect in the leaf adaxial-abaxial pattern, in that these mutants frequently develop trumpet-like leaves and an increased level of *ETT* and *YAB5* [188]. *AN3* expression is detected in all layers of the mesophyll and is not dependent on the function of *AS1* and *AS2* [188]. Furthermore, *AN3* is required for the upregulation of the histone deacetylase genes and a ribosomal protein gene, *RPS27aA* [188]. Thus, the histone deacetylase genes and the ribosomal protein gene may be able to continue to express, because of *AN3* function during intercalary growth to maintain the adaxial-abaxial pattern (and likely also the adaxial-abaxial boundary). Clarifying the mechanism in which the adaxial-abaxial pattern and boundary are maintained by the lamina-growth regulators during lamina growth is an intriguing problem that should be addressed in the future.

## 7. Conclusion and Future Perspectives

In this review, we highlight recent reports that have shown that the leaf adaxial-abaxial boundary is important for both lamina growth and maintenance of the adaxial-abaxial pattern. Among lamina-growth regulators, auxin and the *WOX* genes act at the adaxial-abaxial boundary and are dependent upon the adaxial-abaxial pattern. The function of the *WOX* genes at the adaxial-abaxial boundary is required for preventing the mixing of adaxial and abaxial-specific features. Based on previous studies on adaxial-abaxial patterning and the *WOX* genes, we proposed that the adaxial-abaxial pattern of the outer layer affects that of the inner layers and that the *WOX* genes at the leaf margin are important for the maintenance of the adaxial-abaxial pattern of the outer layer.

In animal development, boundaries help prevent the intermingling of two different cell populations [3]. The integrity of the boundaries is primarily challenged by cell division and/or tissue deformation during embryogenesis or organ growth and is maintained via two different mechanisms (the lineage-based boundary and non-lineage boundary). In both boundaries, the fates of cells remain during organogenesis. When the boundaries receive pressure to deform, straight and smooth boundaries are maintained by their own features, for example, due to increased cell bond tension and decreased cell division rate, in the case of the lineage-based boundary, and due to switching of cell fates based on the positional signal(s), in the case of the non-lineage boundary [3,10,11,12,13]. In contrast, the leaf adaxial-abaxial boundary plays a role in preventing the intermingling of the adaxial and abaxial “features” or “gene expressions”, rather than cell populations. This fact indicates that gene expression patterns dynamics are a major cause of perturbation of the sharp and smooth boundary. The small RNAs, *i.e.*, miR165/166 and ta-siR ARF, are considered to act as potentially mobile signals in leaf primordia and mediate the antagonistic interactions between the adaxial and abaxial regions [19,56]. A mathematical model from our group demonstrates that the antagonistic interaction between two diffusible signaling molecules induces the dynamic pattern of the boundary [165], supporting our concept that the integrity of the boundary is challenged by gene expression dynamics. In fact, the boundary shift is observed in the inner layers during leaf development [165], whereas the position of the boundary appears relatively-stable at the margin during lamina growth. Hence, repression of both the adaxial- and abaxial-specific gene expression by *WOX* genes at the margin would establish a buffer region between the adaxial and abaxial regions to prevent mixing of the adaxial- and abaxial-specific gene expression and features (Figure 5).

In nature, a huge variety in the shape of leaves and flowers is observed, and the molecular basis of the diversity is an intriguing problems. In plant development, the shape of organs is determined by the direction and extent of cell proliferation and cell expansion. The adaxial-abaxial boundary organizes the pattern of cell proliferation in leaf and floral organ primordia and is one of the major causes that determine the shape of lateral organs. Therefore, understanding how the adaxial-abaxial pattern is changed and arranged during the evolution of each plant species is required for understanding the diversity of leaf and flower shapes. Recent reports have demonstrated the alteration of the adaxial-abaxial pattern or *WOX* expression results in the development of lateral organs that are atypical in shape in several species: for example, *PRSb/WOX3* expression in the *de novo* margin of a unifacial leaf of *Juncus prismatocarpus* [190], expansion of the expression domain of the *FIL* orthologous gene in a peltate leaf of *Tropaeolum majus* [191] and the dynamic patterns of *ETT1* and *PHB3* in anthers of *O. sativa* [192]. In conclusion, the analyses of the adaxial-abaxial pattern and boundary in both model and non-model plant species is expected to shed light on the molecular basis of the diversity of leaves and flowers.
